# The Contribution of the Face-Name Associative Recognition Test to Objectifying the Impairment of Associative Memory in Subjective Cognitive Decline

**DOI:** 10.3390/brainsci14111129

**Published:** 2024-11-08

**Authors:** Joël Macoir, Pascale Tremblay, Carol Hudon

**Affiliations:** 1 Faculté de Médecine, École des Sciences de la Réadaptation, Université Laval, Québec, QC G1V 0A6, Canada; pascale.tremblay@fmed.ulaval.ca; 2 Centre de Recherche CERVO—Brain Research Centre, Québec, QC G1J 2G3, Canada; carol.hudon@psy.ulaval.ca; 3 Faculté des Sciences Sociales, École de Psychologie, Université Laval, Québec, QC G1V 0A6, Canada; 4 Centre de Recherche VITAM, Québec, QC G1J 2G1, Canada

**Keywords:** subjective cognitive decline, Alzheimer’s disease, episodic memory, associative memory, assessment

## Abstract

Objective: Subjective cognitive decline (SCD) is defined as a self-reported perception of cognitive decline that occurs without clear objective signs of cognitive impairment. There is still uncertainty in the literature about the reliability of SCD as an accurate indicator of the early stages of major neurocognitive disorders. Furthermore, objectifying cognitive impairment in SCD is difficult, mainly due to the insensitivity of the assessment instruments. The main objective of this study was to investigate the potential contribution of the face-name associative recognition test (FNART) to the objective identification of memory impairment in SCD. Method: A research sample of 69 adults with SCD and 69 healthy controls (HCs) recruited in the community were administered in the FNART, which included 32 photographs of neutral faces associated with 32 first names. Results: The total score of the HC group in the FNART was significantly better than that of the SCD group. Moreover, analyses based on the serial position of the stimuli showed that the SCD group performed significantly worse than the HC group only for the middle items (stimuli placed at the beginning or end of learning lists are more likely to be recalled than those presented in the middle), while no primacy and recency effects were found in the HCs. Conclusions: These findings indicate that associative episodic memory is more vulnerable in individuals with subjective cognitive decline (SCD) compared to those without cognitive complaints. Additionally, they suggest that the FNART may be effective in identifying cognitive decline in the preclinical stage of Alzheimer’s disease.

## 1. Introduction

The Alzheimer’s disease (AD) pathology develops slowly over many years, sometimes decades [1,2]. At the end of this preclinical process, people may have concerns and complaints about their cognitive abilities. This condition, called subjective cognitive decline (SCD), refers to a self-perceived cognitive decline without obvious objective cognitive impairment [3,4]. Objectifying cognitive impairment in SCD is a particular challenge due to the limited sensitivity of neuropsychological assessment tools and the compensatory mechanisms by which those affected often perform normally [3,5].

Concerns about memory or language are often associated with increasing age. Between 50% and 80% of people aged 70 and over report that their cognitive abilities are declining, despite being within normal limits on cognitive tests [6]. In a recent meta-analysis of longitudinal studies investigating the risk of major neurocognitive disorders (MNCD) or mild cognitive impairment (MCI) among individuals with SCD, Pike et al. [7] found a mean prevalence of 44% (with a range of 5–84%) of SCD across 46 studies encompassing over 74,000 participants.

Expressing complaints about cognitive functioning is not trivial, as self-reported subtle cognitive issues have been linked to a higher risk of developing MNCD [8,9]. Nevertheless, despite the available evidence, the literature remains inconclusive as to the reliability of the SCD condition in effectively representing the initial stages of MNCD, particularly when psychiatric symptoms or conditions such as depression are present [10,11].

Identifying neurodegenerative diseases at an early stage is a major focus of public health initiatives and clinical research aimed at preventing MNCD. The predictive value of SCD statuses for MCI and AD has been investigated in various studies, with contrasting results according to the recruitment site and method. In their systematic review comprising 10 studies on this topic, conducted in 4331 individuals with SCD, Parfenov et al. [12] showed that the relative risk of MCI and MNCD conversion in SCD, compared to healthy controls, was 2.15 (95%CI 1.39–3.30; *p* = 0.005) and 2.17 (95%CI 1.53–3.07; *p* < 0.05), respectively. Likewise, in a multicenter study examining SCD across community-based settings and memory clinics with 2978 SCD participants and 1391 controls, Slot et al. [9] found a higher dementia incidence among those with SCD (17.7 per 1000 person-years) compared to controls (14.2 per 1000 person-years). This difference was particularly pronounced in memory clinics compared to community-based settings.

Researchers have also attempted to objectify cognitive impairment in SCD in various cognitive domains. Many of these studies have in common the use of tasks that are more cognitively demanding than standard neuropsychological tests. In the domain of executive functions, studies have shown that participants with SCD perform worse on very demanding verbal fluency tasks, such as action verb fluency [13,14] or alternating and orthographic constraint fluency [15]. Impairment of long-term episodic memory has also been found in SCD in studies of visual recognition memory [16], autobiographical memory [17], memory binding [18], prospective memory [19], and learning of new information [20,21].

The usefulness of the face-name association memory task (FNAMT) in the detection of memory impairment in SCD has been reported in a few studies, with controversial results [22,23]. This task developed by Rentz et al. [24] consists of the encoding of visual (faces of people) and verbal (names) stimuli, followed by their immediate and delayed recall. The FNAMT assesses the cross-modal binding processes of episodic memory involved in the creation of associative links between independent items [25]. In their study, Kormas et al. [22] found that the 21 participants with SCD who were recruited in a memory clinic showed lower associative memory performance on the FNAMT than healthy participants. In contrast, Florez-Vázquez et al. [23] found no difference in the test performance between 35 healthy participants and 37 SCD participants recruited from the community. A similar discrepancy in results was found in studies in which authors examined the relationship between performance on the FNAMT and amyloid-β burden (Aβ) in healthy individuals. Rentz et al. [24] found that higher global Aβ deposition was significantly related to poorer performance on the FNAMT in 45 healthy participants recruited from a memory clinic. Similar results were reported by Sanabria et al. [26], who studied a large group of 200 people with SCD recruited from a memory clinic. In contrast, Mizuno et al. [27] found no correlation between elevated amyloid-β and SCD symptoms, neither in 22 SCD participants recruited from an academic memory clinic nor in 41 healthy participants recruited from the community.

Instead of a test in which participants had to explicitly recall the names associated with the faces, Polcher et al. [21] used the face-name associative recognition test (FNART), an adaptation of the FNAMT in which participants had to recognize the names associated with each face during encoding (i.e., point to the correct rather than the distractor name). The 32 participants with SCD they recruited from a memory clinic showed lower associative memory performance on the FNART compared to healthy participants. As pointed out by Polcher et al. [21], the FNART is easier than the FNAMT and could; therefore, avoid floor effects, such as those found in healthy people in the studies by Alegret et al. [28] (mean values for people aged 65 and over: immediate recall = 2.1/16; delayed recall = 1.9/16) or by Alviarez-Schulze et al. [29] (mean values for people aged 55 to 65 with less than 16 years of formal education: immediate recall = 2.34/16; delayed recall = 2.39/16). In addition, the FNART paradigm is less stressful than the FNAMT, an advantage that is particularly important when the test is used with people who have concerns about their memory abilities [21].

The main objective of this study was to investigate the potential contribution of the FNART to the objective identification of memory impairment in SCD in a larger group of population-recruited participants in whom cognitive complaints were documented using a structured questionnaire. As many older people have concerns about their cognitive abilities [30], despite being within normal limits on cognitive tests, structured questionnaires are considered optimal to objectify actual cognitive complaints [4,31].

Previous research has shown that memory is better for the faces of one’s own age group, an effect known as “own-age bias” [32,33]. In light of this literature, the second objective of our study was to investigate whether own-group bias would be observed when SCD participants are involved in the FNART. Finally, studies have shown that the successful recall of a stimulus is influenced by the order of its presentation within the learning list. Stimuli presented at the beginning or at the end of the list are more likely to be recalled. These phenomena are known as the “primacy effect” and the “recency effect”, respectively [34]. Patients with AD show impaired primacy and intact recency effects [35,36], and it has been shown that a reduced primacy effect observed at the MCI stage can predict the progression of AD [37,38]. Based on this literature, the third objective of our study was to investigate whether primacy and recency effects occur when FNART is administered to SCD participants.

## 2. Materials and Methods

### 2.1. Participants

A research sample of 69 adults with SCD and 69 healthy controls (HCs) was recruited for this study through advertising in the community. The participants, whose mother tongue and current language was French, were between 52 and 81 years old. All individuals with SCD reported concerns about their cognitive abilities and met the criteria outlined by Jessen et al. [5]: (a) a self-reported, ongoing decline in cognitive abilities compared to a previously normal state; (b) this decline is unrelated to an acute event; and (c) performance on standardized cognitive tests—adjusted for age, gender, and education—is within the normal range. To ensure their physical and mental well-being, a self-reported health status questionnaire was administered to all participants.

Participants with specific prior or current medical conditions were ineligible for inclusion in the study. Exclusions encompassed individuals with histories of moderate or severe traumatic brain injury, cerebrovascular disease, delirium within the past six months, intracranial surgery, neurological disorders of cerebral origin, encephalitis or bacterial meningitis, recent oncological treatments within the past 12 months, and general anesthesia within the past six months. Additionally, those with unstable metabolic or medical conditions (e.g., untreated hypothyroidism), current psychiatric disorders as defined by DSM-V (Axis I) criteria [39], alcoholism or substance abuse within the previous 12 months, uncorrected self-reported vision and hearing problems, current use of experimental medications, or inability to provide informed consent were also excluded. The information regarding these exclusion criteria was obtained from self-reports provided by the participants.

All study participants gave written informed consent following the principles of the Declaration of Helsinki. The study received approval from the local research ethics board, specifically the Ethics Committee on Sectoral Research in Neurosciences and Mental Health of the CIUSSS de la Capitale-Nationale (project number 2019-1529).

### 2.2. Clinical Assessment and Group Characterization

To confirm compliance with the inclusion and exclusion criteria and to categorize participants into the SCD and HC groups, all participants underwent a clinical assessment that included an evaluation of depressive and anxiety symptoms, general cognitive functioning, and cognitive complaints. For the latter, the Questionnaire de Dépistage de la Plainte Cognitive (Screening Questionnaire for Cognitive Complaints; QDPC) (Dion et al., unpublished) was used, which is directly aligned with the research criteria for SCD established by Jessen et al. [5]. This user-friendly, simple, and standardized questionnaire addresses an individual’s cognitive decline in comparison to his/her former level of functioning as well as his/her cognitive function compared to others in the same age group. The questions and sub-questions of the QDPC are listed in Table 1.

To address the frequent occurrence of depressive and anxiety symptoms in people with SCD [40,41], all participants were assessed with the 30 item Geriatric Depression Scale (GDS-30) [42] and the Geriatric Anxiety Inventory (GAI) [43]. These instruments consisted of a series of yes-no questions and were specifically designed to identify symptoms of depression and anxiety, respectively. The Montreal Cognitive Assessment (MoCA) [44] was used to assess general cognitive function, specifically targeting cognitive impairment associated with MCI. Despite its demonstrated sensitivity in detecting mild cognitive deficits, several studies [45,46] have indicated that the cutoff score of 26, as suggested by Nasreddine et al. (2005) [44], may lead to an increased risk of false positive results in individuals of older age. Furthermore, Carson et al. [47] conducted a meta-analysis involving 304 studies, concluding that using a MoCA cutoff score of 23 instead of the originally recommended score of 26 lowers the false-positive rate and improves overall diagnostic accuracy. Taking these recommendations into account, we used the regression-based norms that we established for the MoCA test in middle-aged and elderly individuals from the French-Quebec population [48]. As highlighted by Carson et al. [47], these norms, which take into account age, education, and sex, optimize both specificity and sensitivity, thus increasing diagnostic accuracy.

### 2.3. Experimental Task

#### 2.3.1. Material

In this study, we used a FNART adapted from the study by Polcher et al. [21]. Thirty-two photographs of neutral faces were selected from the Minear and Park database [49], evenly distributed between the faces of men (*n* = 16) and women (*n* = 16) and across 4 age categories: 18 to 29 years; 30 to 49 years; 50 to 69 years; 70 to 94 years. Thirty-two two-syllable first names were assigned to each of these photos, selected based on the frequency of their use in Quebec in each of the 4 selected time periods. For people aged 18 to 49, this frequency was determined using the first name bank compiled by Retraite Québec [50]. No data were available for people born before 1980. Therefore, the first names of persons aged 49 and older were selected from information on genealogy websites.

#### 2.3.2. Procedure

**Figure 1:**
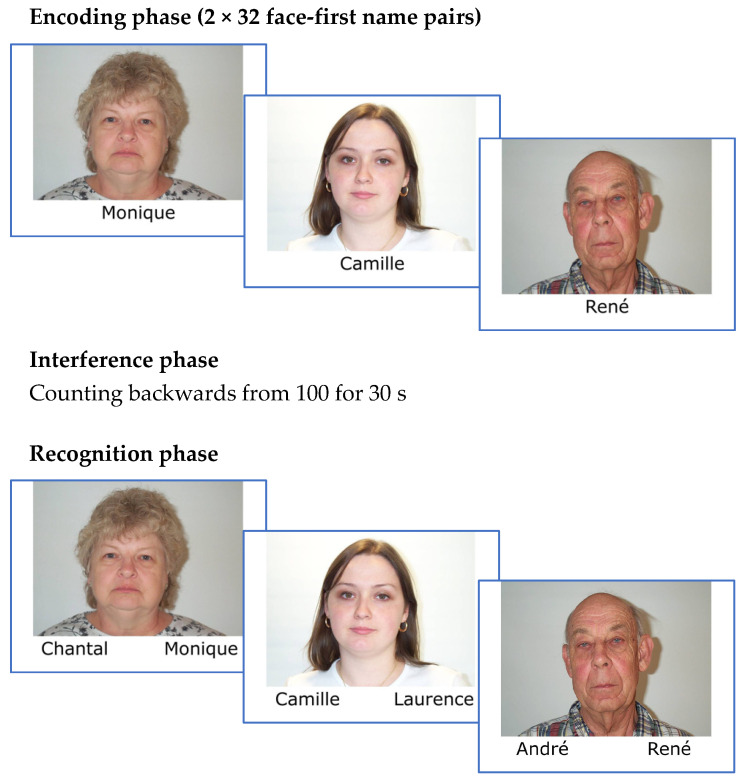
Outline of the three phases of the face-name associative recognition test (faces were selected from the Minear and Park database [49]).

Participants were assessed in a 50 min session in which each of the clinical and cognitive profile characterization tests and the experimental task were administered in the same order. The experimental task was carried out in the following three different phases: encoding, interference, and recognition. Examples of these three phases are shown in Figure 1.

During the encoding phase, each of the 32 “face-first name” pairs was presented on the computer screen for 5 s, twice in two different blocks and in random order. For each pair, the participant was asked to read the first name aloud and to focus on the association between the photo and the name for further retrieval. The participants were given the following instructions in French: “I will show you photos of men’s and women’s faces and their first names one after the other. Each of these photos will be shown twice on the computer screen for 5 s and your task is to read each first name out loud and try to remember the different associations between the face and the first name, i.e., the first name that goes with each face. It is important that you concentrate and try to memorize the association between first names and photos as well as possible, because we will then set you a task in which you have to decide which of the two first names is associated with each photo. Are you ready?”.

During the interference phase, participants were asked to count backwards from 100 for 30 s.

In the recognition phase, each of the 32 faces was presented on the computer screen, together with two first names: the corresponding first name and one of the first names corresponding to another face, taking into account the biological sex and age of the face. The participant’s task was to designate the first name correctly associated with the face without a time limit. Each first name was; therefore, presented twice (once as a target and once as a distractor). The position of the correct first name and the distracter were randomly counterbalanced. The trials were presented in a pseudorandom order, meaning that the same first name cannot be presented two or more times during 6 consecutive trials. The participants were given the following instructions in French: “We will now check whether you have retained the associations between each face and the corresponding first name. On the computer screen, each face from the previous task will be shown again, but this time with two first names, one at the bottom of the right-hand photo and one at the bottom of the left-hand photo. One of these two first names matches the face, while the other does not. Your task is to point to the first name that is correctly assigned to the photo. Do you understand the task? Are you ready?”.

#### 2.3.3. Statistical Analyses

All statistical analyses were carried out using the freely accessible statistical package Jamovi [51].

First, as distributions were not normal, the Mann–Whitney U test (age, educational level, MoCA, GDS, and GAI) was used to compare the groups based on demographic data, except for sex, which was examined with the chi-square test. The scoring method was based on the number of correct responses in the FNART. For the main objective of the study, the total number of correct associations in the FNART was calculated. For the second objective of the study, “own-age bias”, the number of correct answers was calculated for the associations of names with faces of people aged 18 to 29 years (Age 1) and 50 to 69 years (Age 2). For the third objective of the study, “primacy and recency effects”, the number of correct answers was calculated for the associations of names with faces of people who were presented at the beginning (stimuli 1, 2, and 3), in the middle (stimuli 15, 16, and 17) and at the end (stimuli 30, 31, and 32) of the second encoding presentation.

The relation between the sociodemographic and clinical variables and the main dependent variables of the experimental task was examined using Spearman tests. The data distributions of the two groups were checked for skewness using the Shapiro–Wilk test and histograms. In addition, the normality and homoscedasticity of the residuals were checked using suitable visualizations, namely quantile–quantile (Q–Q) plots and plots of the residuals versus the fitted values.

The data distributions for all variables in the FNART were skewed (all skewness values were between −0.177 and −1.97; all kurtosis values were between −0.89 and 3.10; Shapiro–Wilk W was between 0.959 and 0.535; Shapiro–Wilk *p*-values = 0.023 for the total score in the SCD group and =0.001 or <0.001 for all other variables) and the residual plots showed violations of homoscedasticity. Therefore, the nonparametric Mann–Whitney U test was used to compare the two groups. In addition, within-group comparisons for the second (“own-age bias”) and third (“primacy and recency effects”) objectives of the study were computed using nonparametric Friedman repeated-measures ANOVAs and pairwise post hoc Durbin-Conover comparisons between factor levels when an analysis indicated a significant main effect. Effect sizes were evaluated using rank biserial correlation and interpreted as small (0.10–0.29), medium (0.30–0.49), and large (≥0.50) [52].

Finally, we used logistic regressions to assess the additive contribution of the FNART in the classification of SCD and HC. Results were presented as odds ratios with 95% confidence intervals (CIs). We reported the accuracy, sensitivity, and specificity as well as the positive and negative predictive values. The area under the ROC curve (AUROC) was also calculated.

## 3. Results

The demographic data and clinical test results are shown in Table 2. The statistical tests showed that the two groups were equivalent with respect to age and sex; however, the participants with SCD were more educated than the HCs. All participants performed within the normal range on the MoCA based on normative data that considered age, education, and sex [48]. The GDS-30 scores of HCs and participants with SCD ranged from 0 to 16 and 0 to 20, respectively. There was no statistical difference between the two groups, and none of the participants had clinical depression according to DSM-V criteria [39]. There was also no significant difference between the GAI scores of the HCs and the participants with SCD, which ranged from 0 to 17 and 0 to 16, respectively. None of the participants had clinical anxiety according to DSM-V criteria.

Regarding the influence of education level on performance, the analysis showed only a positive correlation with performance on the Age 1 associations (Spearman Rho = 0.229, *p* = 0.007) and performance on the associations presented at the beginning of the second encoding presentation (Spearman Rho = 0.191, *p* = 0.025). In view of these results, educational level was not included as a covariate in the analyses.

Table 3 shows the mean, SD, and range of results for the FNART by group and performance according to the dependent variables. As can be seen from this table, the HC group’s total score on the FNART was significantly better than that of the SCD group. With regard to the own-age bias analysis, Mann–Whitney U-tests showed that the performance of the SCD group was statistically lower than that of the HC group for both associations of names with faces of people aged 18 to 29 years (Age 1) and 50 to 69 years (Age 2). The within-group comparisons revealed no statistical difference between performance for Age 1 and Age 2 faces in the control group (Friedman *χ*^2^ = 2.57, *p* = 0.11) and the SCD group (Friedman *χ*^2^ = 1.85, *p* = 0.17). With respect to the primacy-recency analyses, the performance of the SCD group was only significantly lower than that of the HC group for the middle items (see Table 3). The within-group comparisons revealed significant differences depending on the serial position in the SCD group only (Friedman *χ*^2^ = 8.69, *p* = 0.013). The performance of the SCD participants was lower on the middle items than on the first and last items. The pairwise comparison tests showed a tendency towards significance between the first three items and the middle items (Durbin-Conover statistic = 1.93, *p* = 0.056) and a significant difference between the middle and last items and a significant difference between the middle and the last items (Durbin-Conover statistic = 2.98, *p* = 0.003).

In terms of predicting the classification of participants into the two groups, a comparison of the models with each score using AIC and BIC (see Table 4) yielded the following results: the total score of the FNART correctly identified 52% of the SCD cases (AUC = 0.63; sensitivity = 0.59; specificity = 0.52); the score for the associations of names with faces of people aged 18 to 29 years (Age 1) correctly identified 52% of the SCD cases (AUC = 0.62; sensitivity = 0.64; specificity = 0.52); the score for the associations of names with faces of people aged 50 to 69 years (Age 2) correctly identified 61% of the SCD cases (AUC = 0.60; sensitivity = 0.565; specificity = 0.61); the score for the associations of names with faces for the first three items of the list correctly identified 41% of the SCD cases (AUC = 0.51; sensitivity = 0.64; specificity = 0.41); the score for the associations of names with faces for the middle items of the list correctly identified 54% of the SCD cases (AUC = 0.615; sensitivity = 0.71; specificity = 0.54); the score for the associations of names with faces for the last three items of the list correctly identified 30% of the SCD cases (AUC = 0.54; sensitivity = 0.78; specificity = 0.30). According to these results, the variable that best identified SCD was the score for the association of names with faces of people aged 50 to 69 years.

## 4. Discussion

The main objective of the present study was to investigate the potential contribution of the FNART to the identification of memory impairment in SCD. As in the study by Polcher et al. [21], the only other study in which the FNART was used in SCD, we found that the performance of the participants without cognitive complaints was significantly better in the FNART than that of the SCD group. While the SCD participants in the study by Polcher et al. [21] were recruited from a memory clinic, the participants in the present study came from the community. The results of the few studies that have compared cognitive and psychiatric symptoms and the pattern of structural changes in the brain indicate that SCD samples from the general population and from memory clinics are not similar [53,54]. In their recent literature review of the results of structural imaging in SCD, Pini and Wennberg [55] analyzed the studies that examined alterations in brain regions typically seen in AD. They found that nine of the eleven studies reported AD-typical atrophy in SCD participants recruited from the community, while no atrophy of these regions was reported in about one-third of the studies conducted with SCD participants recruited from memory clinics who, instead, showed a complex and heterogenous pattern of structural alterations in the brain. The greater heterogeneity of the clinic compared to the community SCD group was also found in studies on psychiatric symptoms. Espenes et al. [56], for example, found that depressive symptoms are more prevalent in people with SCD who seek medical help than in people who do not seek help. Moreover, SCD individuals with few depressive symptoms tend to have a smaller hippocampus than those with more depressive symptoms [57], a clinical feature that might be associated with a higher risk of dementia [58]. The participation of community-dwelling participants in this study, therefore, strengthens the interpretation of our results as an actual objectification of cognitive decline in SCD.

The second objective of our study was to investigate whether own-group bias would be observed when administering the FNART to SCD participants. Many studies have shown that recognition memory for faces of one’s own age group is superior to memory for faces of other age groups [32,33]. In neurodegenerative diseases, only one study reported the preservation of the own-age bias in 20 individuals with AD [59]. In this study, the results showed no differences between the two groups in the total number of correct associations of names with faces of people aged 18–29 years (Age 1) and 50–69 years (Age 2). It has been shown that the own-age bias in face recognition might depend on the requirements of the task [60]. Therefore, the absence of own-age bias in the SCD participants in the present study could be due to the nature of the FNART (i.e., memorizing faces and names), whereas the protocols used in the own-age bias studies consisted mainly of processing individual faces (e.g., judging whether or not a face was present in the learning list). Another explanation could come from the results of several studies that have shown that the processing of faces is very heterogeneous and can vary depending on the extent of experience with people of other age groups [61,62].

The third objective of our study was to investigate whether primacy and recency effects occur when the FNART is administered to SCD participants. It is widely recognized that the effective recall of a stimulus depends on the order of its presentation within the learning sequence (beginning, middle, or end of the list) [34,63]. Stimuli positioned at the beginning [64] or at the end [65] of the learning list are generally more likely to be recalled than those presented in the middle, a phenomenon known as primacy and recency effects, respectively. The primacy effect has been explained by rehearsal, i.e., the items presented in the first positions are remembered better because more time has been spent encoding them during the task [66] or because they receive more attentional resources [67]. In contrast, the recency effect occurs because the last items of the list are still maintained in working memory and can; therefore, be recalled more easily than the items in the middle of the list [68,69]. In AD, patients typically show a reduced primacy effect, while the recency effect remains largely normal [70,71,72]. In MCI, a recent literature review also showed reduced primacy and intact recency effects [36]. Moreover, it has been shown that a reduced primacy effect observed at the MCI stage can predict the progression of AD [37,38].

In this study, the only difference between the two groups was found in the middle items, where the SCD participants were less able than the HC participants to correctly identify the first names associated with the faces. The objectification of serial position effects in healthy participants was inconclusive. However, participants with SCD showed a clear recency effect and a trend toward significance for the primacy effect. The comparison of these results with previous studies is risky as most studies have investigated the serial position effect using a free recall paradigm. Primacy and recency effects were found in adults with a similar association paradigm to that used in the present study [73]. Associative memory tasks provide better control of retrieval processes than free recall tasks, and further studies are needed to investigate their utility in objectifying differences in serial position effects between healthy and pathologically aging participants.

The present study has limitations. First, its cross-sectional design prevented the tracking of cognitive decline over time in individuals with subjective cognitive decline (SCD), making it impossible to evaluate how performance on the FNART might predict the progression from SCD to MCI or AD. Second, the sampling method used in the study represents another limitation, as it is a common source of variability in the results of studies on SCD [74]. Studies have shown that participants with SCD recruited from memory clinics are more likely to develop MCI compared to participants recruited from the general population [53,75]. However, as Rodríguez-Gómez et al. [74] noted, community-based samples provide a more accurate representation of individuals with cognitive complaints. As participants with SCD had different demographic and neuropsychological characteristics depending on the recruitment method [76], further studies aiming to objectify cognitive impairment in SCD should include and compare participants recruited from the community and from memory clinics.

In conclusion, identifying actual cognitive impairment in SCD is challenging, particularly because of the presence of confounding factors such as depression and anxiety, but also because of the lack of sensitivity of many neuropsychological tests to mild but significant difficulties. This justifies the development of new quasi-ecological tests, such as the FNART, which are well adapted to the early symptoms and underlying pathological causes of cognitive decline. The binding process that characterizes FNART is of particular interest for clinical studies on AD because the new associations created in episodic memory are known to rely on hippocampal and parahippocampal regions [77,78]. These structures are affected first and foremost in AD pathology [79,80], including MCI [81,82]. Moreover, studies have also shown structural and functional changes in the hippocampus and parahippocampus in participants with SCD [83,84]. Associative memory has been found to be especially sensitive to the early stages of AD [85]. This is particularly true when it comes to cross-modal associations such as faces and names [20,86]. This reinforces the idea of using simple-to-administer tests, which are known to target specific brain structures, to objectify memory impairment in SCD. While the FNART alone cannot classify participants with SCD, the findings of this study enhance our clinical understanding of the condition. In SCD, cognitive domains that are usually impacted in MCI, such as associative episodic memory, may decline more significantly than what is generally seen in the broader population.

## Figures and Tables

**Table 1:** Screening questionnaire for cognitive complaints.

Questions
1. Are you worried about how your memory is working? 2. Do you think your memory has changed in the last 10 years? 2.1. If yes, how long have you observed a decline in memory functioning? 3. Do you feel that your memory is worse than that of other people your age? 3.1. If yes, and it is worse, do you feel that you have always had a poorer memory than other people your age? 3.2. If no, and it is the same would you say that, in the past: 3.2.1. your memory was at the same level as most other people your age? 3.2.2. your memory was better than most other people your age?
**Categorization of SCD**
Answer YES to questions 2 and 3, and YES to subquestion 3.1 Answer YES to question 2, No to question 3 but YES to subquestion 3.2.2

**Table 2:** Demographic and clinical characteristics of the two groups.

	HC (*n* = 69)		SCD (*n* = 69)				
	M (SD)	Min–Max	M (SD)	Min–Max	*t*/*U*	*p*	Effect Size
Age	65.6 (6.88)	52–80	66.8 (5.34)	55–80	1.13	0.26	*d* = 0.193
Education	15.9 (3.47)	8–23	17.7 (2.98)	11–23	3.34	0.001	*d* = 0.569
Males/females	26/43		32/37		1.07 ^t^	0.30	
MoCA (30)	27.3 (1.62)	23–30	26.8 (2.06)	22–30	2000	0.10	*rbc* = 0.160
GDS (30) ^τ^	7.05 (4.48)	0–16	8.00 (4.39)	0–20	1.22	0.23	*d =* 0.214
GAI (20) ^λ^	3.40 (3.58)	0–17	4.56 (4.71)	0–16	1903	0.335	*d =* 0.097

Note: *d* = Cohen’s *d*; GAI = Geriatric Anxiety Inventory; GDS = Geriatric Depression Scale; HC = healthy controls; M = Mean; min–max = minimal–maximal test score value; MoCA = Montreal Cognitive Assessment; *rbc* = rank-biserial correlation; SCD = subjective cognitive decline; SD = standard deviation. ^t^ = Pearson’s Chi-squared test. ^τ^ missing data for the GDS = 8 HC, 1 SCD; ^λ^ missing data for the GAI = 7 HC, 1 SCD.

**Table 3:** Results on the FNART by group, performance, age, and serial position of the face-name associations.

	HC (*n* = 69)			SCD (*n* = 69)					
Performance	Mean	SD	Range	Mean	SD	Range	* U *	*p*	Effect Size
Total response	27.4	3.62	17–32	25.6	4.28	16–32	1782	0.011 *	0.2516
**Associations/Age**									
Age 1 (18–29 years)	6.8	1.29	3–8	6.49	1.35	3–8	1797	0.012 *	0.245
Age 2 (50–69 years)	6.48	1.55	0–8	6.19	1.50	3–8	1921	0.048 *	0.193
**Associations/Position**	**Mean**	**SD**	**Range**	**Mean**	**SD**	**Range**			
Initial (items 1-2-3)	2.54	0.68	1–3	2.52	0.655	0–3	2322	0.77	0.025
Middle (items 15-16-17)	2.58	0.74	0–3	2.29	0.77	0–3	1834	0.008 **	0.23
End (items 30-31-32)	2.74	0.53	1–3	2.65	0.56	1–3	2183	0.27	0.083

Note: HC: healthy controls; SCD: subjective cognitive decline; SD: standard deviation; effect size expressed by rank-biserial correlation. * *p* < 0.05; ** *p* < 0.01.

**Table 4:** Logistic regression models for prediction of groups HC vs. SCD using the results of experimental tasks in separate model.

**HC vs. SCD**	**95% CI for Odds Ratio**						
**Variables**	**Estimate (SE)**	**Lower**	**OR**	**Upper**	***p***	**AIC**	**BIC**
Total response ^£^	0.115	1.027	1.12	1.225	=0.01 **	188	194
Associations/Age							
Age 1 (18–29 years) ^≠^	0.197	1.035	1.22	1.434	=0.017 *	189	195
Age 2 (50–69 years) ^¥^	0.161	1.0145	1.175	1.361	=0.03 *	190	196
Associations/position							
Initial (items 1-2-3) ^κ^	0.033	0.624	1.03	1.71	=0.89	195	201
Middle (items 15-16-17) ^λ^	0.521	1.06	1.68	2.28	=0.03 *	190	196
End (items 30-31-32) ^μ^	0.294	0.72	1.34	2.49	=0.35	194	200

Note: AIC: Akaike information criterion; BIC: Bayesian information criterion; CI = confidence interval; HC: healthy controls; OR = odds ratio; SCD: subjective cognitive decline; SE = standard error. * *p* < 0.05; ** *p* < 0.01. ^£^: Model *χ*^2^ = 6.97, *p* < 0.01, Deviance = 184, *R*^2^ = 0.0365 (McFadden), 0.0493 (Cox and Snell), 0.066 (Nagelkerke). ^≠^: Model *χ*^2^ = 6.07, *p* = 0.014, Deviance = 185, *R*^2^ = 0.032 (McFadden), 0.043 (Cox and Snell), 0.057 (Nagelkerke). ^¥^: Model *χ*^2^ = 4.84, *p* < 0.03, Deviance = 186, *R*^2^ = 0.025 (McFadden), 0.0345 (Cox and Snell), 0.046 (Nagelkerke). ^κ^: Model *χ*^2^ = 0.02, *p* < 0.9, Deviance = 191, *R*^2^ = 8.66 × 10^−5^ (McFadden), 1.20 × 10^−4^ (Cox and Snell), 1.60 × 10^−4^ (Nagelkerke). ^λ^: Model *χ*^2^ = 5.11, *p* < 0.02, Deviance = 186, *R*^2^ = 0.027 (McFadden), 0.036 (Cox and Snell), 0.048 (Nagelkerke). ^μ^: Model *χ*^2^ = 0.88, *p* < 0.35, Deviance = 190, *R*^2^ = 0.005 (McFadden), 0.006 (Cox and Snell), 0.008 (Nagelkerke).

## Data Availability

The data presented in this study are available on request from the corresponding author due to ethical restrictions.
